# App-Based Interventions for Moderate to Severe Depression: A Systematic Review and Meta-Analysis

**DOI:** 10.1001/jamanetworkopen.2023.44120

**Published:** 2023-11-20

**Authors:** Hayoung Bae, Hyemin Shin, Han-Gil Ji, Jun Soo Kwon, Hyungsook Kim, Ji-Won Hur

**Affiliations:** 1 School of Psychology, Korea University, Seoul, Republic of Korea; 2 Graduate School of Culture Technology, Korea Advanced Institute of Science and Technology, Daejeon, Republic of Korea; 3 Department of Psychiatry, Seoul National University College of Medicine, Seoul, Republic of Korea; 4 Graduate School of Public Policy, Hanyang University, Seoul, Republic of Korea; 5 Hanyang Digital Healthcare Center, Hanyang University, Seoul, Republic of Korea

## Abstract

**Importance:**

Mobile mental health applications (apps) for moderate to severe depression are proliferating, likely owing to their capacity to overcome the limitations of conventional psychotherapy, but research on the potential moderators of treatment efficacy is lacking.

**Objective:**

To examine the treatment efficacy associated with mobile app interventions for moderate to severe depression and identify the potential moderators associated with better treatment outcomes.

**Data Sources:**

PubMed, Embase, and PsycINFO were searched from their inception to January 22, 2023.

**Study Selection:**

Only randomized clinical trials evaluating mobile app treatments in adults with moderate to severe depression that published their results in English were included in the analysis.

**Data Extraction and Synthesis:**

Three independent researchers extracted and assessed relevant studies, their risk of bias, the characteristics of the population and study design, and the components of the intervention program following the Preferred Reporting Items for Systematic Reviews and Meta-analyses reporting guidelines. A fixed-effects model was used for data analysis, and exploratory post hoc meta-regression and subgroup analyses were also conducted. Data were analyzed from February 16 to March 25, 2023.

**Main Outcomes and Measures:**

The main outcome was changes in depression symptom severity from before to after treatment, measured by standardized depression assessment instruments. Secondary outcomes included study-, intervention-, and patient-level factors associated with app efficacy.

**Results:**

Of 2128 studies identified, 13 studies evaluating 16 intervention apps with 1470 participants with moderate to severe depression were included in the analysis. The overall pooled effect size of mobile app interventions vs both active and inactive control groups was 0.50 (95% CI, 0.40 to 0.61). Interventions with in-app notifications were associated with significantly lower treatment outcomes (standardized mean difference [SMD], 0.45; 95% CI, 0.29-0.60) than interventions without (SMD, 0.71; 95% CI, 0.54-0.87; *P* = .02). In addition, app interventions delivered for less than 8 weeks were associated with a significantly greater effect size (SMD, 0.77; 95% CI, 0.59-0.96) than interventions delivered for 8 weeks or longer (SMD, 0.43; 95% CI, 0.30-0.57; *P* = .004).

**Conclusions and Relevance:**

In this systematic review and meta-analysis, the feasibility and efficacy of mobile app interventions were supported in treating moderate and severe depression, and practical implications were also provided for developing effective app-based interventions in clinical practice.

## Key Points

**Question:**

What patient characteristics are associated with benefiting from use of mobile application (app) interventions for depression, and under what circumstances?

**Findings:**

This systematic review and meta-analysis of 13 randomized clinical trials of app interventions with 1470 participants found a significant medium effect size for moderate to severe depression, with some variation in effect sizes depending on the characteristics of the population and study design and the components of the intervention program.

**Meaning:**

These findings underscore the efficacy of mobile app interventions for moderate and severe depression both as standalone interventions and adjuncts to conventional treatments and provide confidence that refining the design of intervention programs can further enhance their effectiveness.

## Teaser

This systematic review and meta-analysis examines the treatment efficacy associated with mobile application (app) interventions for moderate to severe depression and identifies factors associated with better treatment outcomes.

## Introduction

Depression remains a significant global health concern and is a leading cause of disability worldwide, affecting an estimated 3.8% of the world population.^1^ On the spectrum of depression, mild depression may manifest as a minor impairment,^2^ while moderate or severe depression is characterized by a more pervasive depressed mood, anhedonia, feelings of worthlessness, excessive guilt, and even suicidal ideation and behavior.^3,4^ The prevalence and alarming symptoms of moderate and severe depression highlight the importance of more applicable evidence-based practices specifically targeting these conditions.

For decades, research has consistently demonstrated the benefits of evidence-based psychotherapy for depression.^5,6,7,8,9^ Unfortunately, a substantial portion of the population with mental disorders still lacks access to needed mental health care. Structural barriers, such as disproportionate distribution of treatment resources and lack of financial affordability, along with attitudinal barriers, such as fear of stigmatization and lack of awareness of treatment effectiveness, contribute to the challenges of mental health care delivery.^10,11^ A World Health Organization World Mental Health Survey Initiative study^12^ found that only 13.7% of people with mental disorders in low- and middle-income countries received relevant treatments, compared with 36.8% of people in high-income countries. These disparities in treatment rates underscore the need to ensure equitable access to evidence-based interventions for populations with mental health problems.

Amidst the challenges posed by the COVID-19 pandemic impeding conventional therapies and the upsurge of technological advances in internet devices, internet-based interventions for mental disorders have gained prominence due to their enhanced efficacy, scalability, and accessibility.^13,14,15^ Among internet-based interventions, mobile health (mHealth) interventions, ie, medical and public health practices supported by mobile, wireless devices,^16^ offer greater freedom with regard to time and distance with the use of portable devices and fewer technical complications compared with other internet-based therapies.^17,18^ However, previous meta-analyses on app-based interventions for depression have reported variable effect sizes, ranging from small^19,20,21^ to medium^22,23,24^ to large.^25^ Notably, a 2022 systematic review and meta-analysis^26^ reported significant symptom reductions associated with app interventions for anxiety but not for depression. Furthermore, research on the efficacy of mobile app interventions, particularly for treating moderate and severe depression, is scarce despite their urgency in treatment. In addition, limited research has explored the factors influencing better treatment outcomes, with some studies yielding inconsistent findings.^20,22,27^ These findings call for further investigation into the efficacy and potential factors that could enhance or attenuate the effectiveness of app-based treatments for moderate to severe depression.

Therefore, this systematic review and meta-analysis aimed to examine the efficacy and associated variables of app-based interventions for depression. We limited the depression severity to moderate and severe depression to avoid any influence of sample heterogeneity on treatment responsiveness. In addition, we sought to scrutinize the population and study design characteristics and components of the intervention program that were associated with better treatment outcomes, using the factors identified from previous research.^21,22,26^

## Methods

This systematic review and meta-analysis followed the Preferred Reporting Items for Systematic Reviews and Meta-analyses (PRISMA) reporting guideline.^28^ We registered the study protocol in PROSPERO on December 3, 2022 (PROSPERO identification No. CRD42022373969). The protocols of all included studies were approved by their institutional review board or ethics committees, and all participants were reported to have provided written or verbal informed consent, with the exception of 1 study^29^ in which the receipt of consent was not explicitly mentioned.

### Search Strategy and Eligibility Criteria

A systematic literature search was conducted on PubMed, Embase, and PsycINFO from inception to January 22, 2023. Search strategy and terms were developed with the participants, intervention, comparison, outcome, and study design (PICOS) framework^30^ and combining Medical Subject Heading terms following 3 concepts: depression, mobile applications, and randomized clinical trials (RCTs) (eMethods 1 in Supplement 1). We included studies if they (1) included participants with unipolar depression aged 18 years or older with a formal diagnosis of depressive disorder or at least moderate depression indicated on validated rating scales, (2) evaluated the efficacy of therapeutic interventions delivered via a mobile app on smartphones and/or tablets, (3) measured depression symptom changes using standardized depression assessment instruments, (4) were RCTs, and (4) were published in English (eMethods 2 in Supplement 1).

### Study Selection and Data Extraction

Three independent researchers (H.B., H.S., and H.-G.J) conducted the literature search and data extraction, while a fourth researcher (J.-W.H). adjudicated unresolved disagreements through discussion. We removed irrelevant articles or duplicates after reading the titles and abstracts and further screened the full texts of the remaining articles. If a study tested the efficacy of multiple apps, both apps were included in the analysis as intervention apps. If studies used more than 1 depression scale as an outcome measure, the primary outcome measures were pooled for review. We also contacted the authors of studies with missing or partially reported outcomes to obtain the necessary information.^31,32,33^

### Quality Assessment

The quality of each included study was assessed using the revised Cochrane Risk of Bias tool (ROB 2.0) against 5 bias domains.^34^ Two independent researchers (H.B. and H.-G.J.) assessed each criterion and discussed disagreements until a consensus was reached. The overall ROB was evaluated as low, with some concerns, or high. Publication bias was assessed using a funnel plot, Egger regression test,^35^ and Duval and Tweedie trim-and-fill analysis.^36^

### Statistical Analysis

Hedge *g* effect sizes were assessed by standard mean differences (SMDs) and 95% CIs to compare treatment efficacy. *I*^2^ values were calculated for the degree of heterogeneity, with 25% indicating low; 50%, moderate; and 75%, high heterogeneity.^37^ A fixed-effects model was used, and sensitivity analyses were conducted using the leave-one-out method.

Preplanned subgroup analyses were performed for studies using active vs inactive controls, studies without a high ROB, and studies using intention-to-treat (ITT) analyses. In addition, exploratory post hoc meta-regression and subgroup analyses were conducted to examine variables moderating the efficacy of mobile app interventions concerning population and study design characteristics and components of the app-based intervention program. We considered 2-sided *P* < .05 statistically significant and used Excel (Microsoft) and Stata MP version 17 (StataCorp) software for all analyses. Data were analyzed from February 16 to March 25, 2023.

## Results

### Study Selection

**Figure 1.**
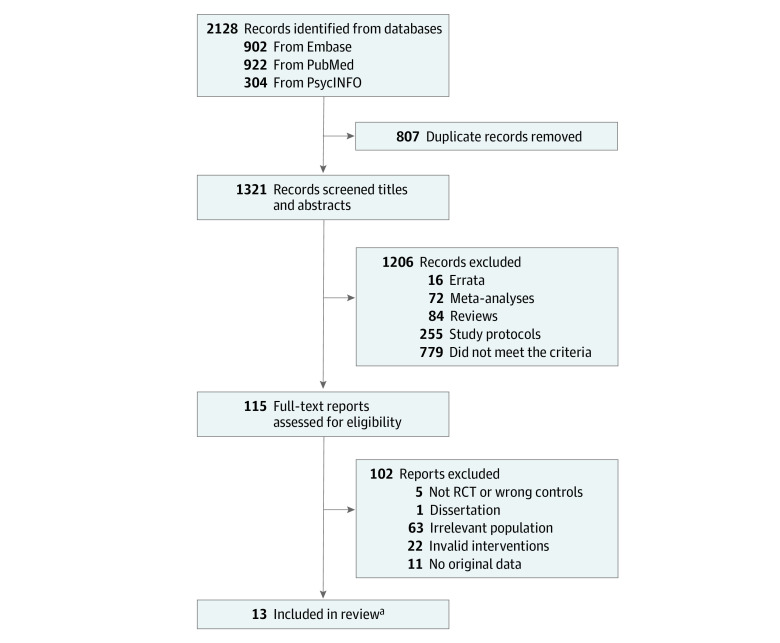
Flowchart of the Study Selection Process RCT indicates randomized clinical trial. ^a^Three studies yielded 2 intervention-control comparisons; hence 16 comparisons were included in the meta-analysis.

A total of 2128 studies were identified from the prespecified databases. After removing 807 duplicates, 1321 studies were screened using titles and abstracts, and 115 studies were identified for full-text screening. With the exclusion of 102 irrelevant studies, 13 studies^29,31,32,33,38,39,40,41,42,43,44,45,46^ evaluating 16 intervention apps were included in the analysis (Figure 1).

### Characteristics of the Included Studies

The 13 studies included a total of 1470 participants (756 in intervention groups and 714 in control groups) and were published between 2017 and 2021. Three studies were conducted in the United States,^31,32,38^ 3 in Europe,^33,44,45^ 6 in Asia,^39,40,41,42,43,46^ and 1 in Australia.^29^ Six studies used waitlist controls,^29,38,39,40,42,46^ while 7 studies used active controls.^31,32,33,41,43,44,45^ The duration of the app interventions varied from 3 to 24 weeks, except for 1 study,^44^ in which the duration was at the discretion of the therapist. In all but 1 study,^44^ participants were screened by a formal clinician diagnosis or meeting the minimum cutoff score on validated depression scales. We included the 1 exception^44^ in the analysis because participants’ depression severity, as assessed by the validation study of the Major Depression Inventory,^47^ was in the severe range (Table 1).

**Table 1.** Characteristics of the Studies Selected for Inclusion

Source	Country	Population characteristics (depression cut-off)	Intervention	Control	Intervention/control				Study duration
					Sample size, No.	Mean age, mean (SD), y	Baseline depression measure, mean (SD)	Dropout rate, %	
Chan et al,^39^ 2023	Hong Kong	Patients with MDD diagnosis and comorbid insomnia (PHQ-9 ≥10)	proACT-S (self-help CBT-I)	Waitlist control	167/153	27.28 (7.25)/27.26 (7.22)	CES-D: 36.88 (8.06)/37.78 (7.8)	34.7 vs 5.9	6 wk
Dahne et al,^32^ 2019	United States	Adults in primary care with elevated depressive symptoms (PHQ-8 >10)	Moodivate (self-help brief BA)	TAU	24/9	44.67 (13.95)/43.11 (11.88)	BDI-II: 28.08 (7.83)/32.33 (13.03)	8.3 vs 0	8 wk
			Moodkit (CBT-based)	TAU	19/9	43.00 (13.63)/43.11 (11.88)	BDI-II: 28.63 (11.30)/32.33 (13.03)	5.3 vs 0	8 wk
Dahne et al,^31^ 2019	United States	Latinx adults with limited English proficiency and elevated depression symptoms (PHQ-8 ≥10; BDI-II >13)	Aptívate! (self-help Spanish-language brief BA)	TAU	22/11	32.68 (9.18)/39.09 (12.07)	BDI-II: 32.18 (9.22)/31.73 (12.27)	4.5 vs 9.1	8 wk
			iCouch CBT (Spanish-lanuage, based on CBT)	TAU	9/11	40.56 (14.16)/39.09 (12.07)	BDI-II: 28.33 (7.71)/31.73 (12.27)	0 vs 9.1	8 wk
Guo et al,^40^ 2020	China	People living with HIV and ≥moderate depression (CES-D ≥16)	Run4Love (based on adapted CBSM and physical activity promotion)	Waitlist control	150/150	28.0 (5.8)/28.6 (5.9)	CES-D: 23.9 (6.4)/24.3 (6.9)	7.3 vs 10	12 wk
Hur et al,^41^ 2018	Korea	People with a diagnosis of Other Specified Depressive Disorders based on *DSM-5*	Todac Todac (scenario-based mind growth based on CBT)	Mood-charting app	17/17	24.76 (3.70)/22.65 (2.42)	BDI-II: 22.65 (7.94)/25.59 (7.93)	29.2 vs 29.2	3 wk
Kageyama et al,^42^ 2021	Japan	People with subthreshold depression (CES-D ≥16)	SPSRS (based on verbal stimulation from positive language)	Waitlist control	16/16	20.00 (0.82)/20.13 (1.59)	CES-D: 20 (4.18)/18.44 (3.52)	0 vs 0	5 wk
Mantani et al,^43^ 2017	Japan	People with antidepressant-refractory MDD diagnosis based on *DSM-5*	Kokoro (based on CBT) with antidepressant switch	Antidepressant switch only	60/57	40.1 (9.0)/41.2 (8.6)	PHQ-9: 13.4 (5.6)/12.6 (5.5)	1.7 vs 0	8 wk
O’Toole et al,^44^ 2019	Denmark	People at risk of suicide, typically with moderate depression^a^	LifeApp’tite (numerous functions, eg, psychoeducation, building safety plan and digital hope kit, and problem solving and acceptance) with TAU	TAU (psychotherapy based on CAMS)	60/69	28.1 (9.2)/29.3 (9.7)	MDI, 34.5 (8.3)/32.9 (9.2)	56.7 vs 43.5	At the discretion of the therapist; typically >8 wk
Raevuori et al,^45^ 2021	Finland	University students with MDD diagnosis based on *ICD-10*	MHP (comprehensive therapist-guided for depression) with TAU	TAU	63/61	24.5 (3.4)/25.8 (5.4)	PHQ-9: 12.44 (4.2)/11.56 (4.6)	30.2 vs 21.3	8 wk
Stiles-Shields et al,^38^ 2019	United States	Participants with moderate depression (PHQ-9 ≥10, QIDS ≥11)	Boost Me (based on BA)	Waitlist control	10/10	Not mentioned	PHQ-9: 15.20 (5.49)/16.1 (3.76)	0 vs 0	intervention: 6 wk; control: 10 wk
			Thought Challenger (based on CT)	Waitlist control	10/10	Not mentioned	PHQ-9: 17.00 (4.62)/16.1 (3.76)	30 vs 0	intervention: 6 wk; control: 10 wk
Tighe et al,^29^ 2017	Australia	Indigenous Australian youth with suicidal ideation and moderate depression (PHQ-9 >10)	Ibobbly (ACT-based suicide prevention)	Waitlist control	31/30	27.48 (9.54)/24.97 (6.28)	PHQ-9: 15.2 (4.5)/15.1 (3.9)	6.5 vs 0	6 wk
Tønning et al,^33^ 2021	Denmark	People with unipolar depressive disorder based on *ICD-10*	Monsenso (symptom monitoring with a feedback loop and CBT) with TAU	TAU	59/61	44.5 (14.0)/43.4 (14.3)	HDRS-17: 14.5 (5.77)/13.9 (6.26)	30.5 vs 36.1	24 wk
Wong et al,^46^ 2021	Hong Kong	People with ≥moderate depressive symptoms (PHQ-9 ≥ 10)	Lifestyle Hub (based on transtheoretical model)	Waitlist control	39/40	36.4 (13.5)/29.7 (10.6)	PHQ-9: 13.5 (4.00)/12.3 (3.6)	12.8 vs 12.5	8 wk

Abbreviations: ACT, acceptance and commitment therapy; BA, behavioral activation; BDI-II, Beck Depression Inventory-II; CAMS, Collaborative Assessment and Management of Suicidality; CBSM, cognitive behavioral stress management; CBT, cognitive behavioral therapy; CBT-I, cognitive behavioral therapy for insomnia; CES-D, Center for Epidemiologic Studies Depression Scale; CT, cognitive therapy; *DSM-5*, *Diagnostic and Statistical Manual of Mental Disorders* (*Fifth Edition*); HDRS-17, Hamilton Depression Rating Scales, 17-item version; *ICD-10*, *International Statistical Classification of Diseases and Related Health Problems, Tenth Revision*; MDD, major depressive disorder; MDI, Major Depression Inventory; MHP, Meru Health Program; PHQ-9, Patient Health Questionnaire, 9-item version; QIDS, Quick Inventory of Depressive Symptoms; SPSRS, Subliminal Priming with Supraliminal Reward Stimulation; TAU, treatment as usual.

^a^ The mean baseline depression scores assessed by the MDI for both intervention and control groups indicated severe symptom severity.^47^

### Quality Assessment

Of 13 included studies, 6 studies^29,32,39,40,41,44^ had a high ROB and 7 studies^31,33,38,42,43,45,46^ demonstrated some concerns of ROB (eFigure 1 and eFigure 2 in Supplement 1). Major sources of bias were the use of self-report questionnaires for outcome measures and the complications of using the double-blind trials. The leave-one-out method showed that the overall effect size was maintained as medium and not notably influenced by a single study, suggesting the robustness of the result (eFigure 3 in Supplement 1).

### Overall Association of the App-Based Interventions With Depression

**Figure 2.**
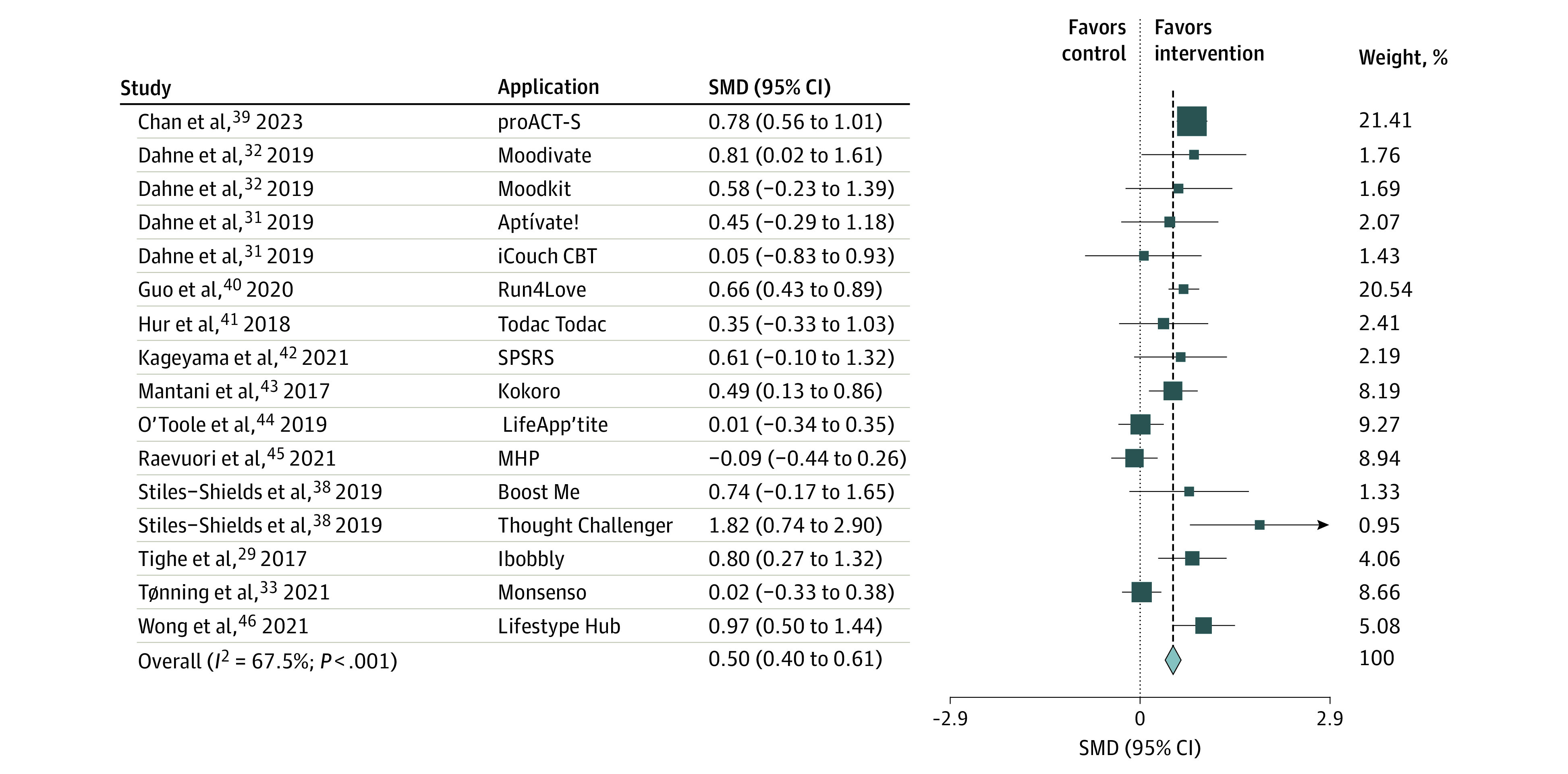
Mobile App Interventions Compared With Controls for the Treatment of Moderate to Severe Depressive Symptoms Size square indicates sample size of each study; SMD, standard mean difference.

A fixed-effects meta-analysis showed that mobile app interventions were associated with significantly reduced depressive symptoms, with a medium effect size (SMD, 0.50; 95% CI, 0.40 to 0.61) and substantial heterogeneity (*Q* = 46.18; *P* < .001; *I*^2^ = 67.5%) (Figure 2). Visual inspection of the funnel plot suggested potential small-study effects (eFigure 4 in Supplement 1). However, Egger test ascertained the absence of publication bias (eTable 1 in Supplement 1), and the trim-and-fill analysis also showed a reduced but small to medium effect size (SMD, 0.43; 95% CI, 0.31 to 0.64), maintained after imputation of 2 studies (eFigure 5 in Supplement 1).

Preplanned subgroup analyses showed a significant association of app interventions with reduction in depressive symptoms compared with active and inactive controls, but the effect size was significantly larger compared with inactive controls (*Q* = 29.76; *P* < .001) (Table 2). Subgroup analysis of the studies using ITT analyses showed a similar effect size (SMD, 0.49; 95% CI, 0.38 to 0.61), while studies with only some ROB concerns showed reduced, small to medium effects size (SMD, 0.35; 95% CI, 0.18 to 0.51) (Table 2).

**Table 2.** Preplanned Subgroup Analyses Based on Study Characteristics

Characteristic	Apps, No.	Sample size, intervention/control, No.	Meta-analysis		Heterogeneity			Between-group tests	
			Hedge *g* (95% CI)	*P* value	*Q*	*P* value	*I* ^2^	*Q*	*P* value
Used active controls	9	333/305	0.175 (0.017-0.333)	.03	10.99	.20	27.2	29.76	<.001
Used inactive controls	7	423/409	0.765 (0.624-0.906)	<.001	5.43	.49	0.0		
Some ROB concerns	9	288/277	0.345 (0.176-0.514)	<.001	25.43	.001	68.5	5.46	.02
High ROB	7	468/437	0.603 (0.468-0.738)	<.001	15.29	.02	60.8		
ITT analysis	10	655/647	0.494 (0.383-0.605)	<.001	38.58	<.001	76.7	0.24	.62
PP analysis	6	101/67	0.581 (0.253-0.909)	.001	7.36	.20	32.1		

Abbreviations: ITT, intention-to-treat; PP, per-protocol; ROB, risk of bias.

### Subgroup Analyses of Population Characteristics

A subgroup analysis of 3 studies that recruited participants who belonged to marginalized groups (ie, patients living with HIV,^40^ US-resident Latinx adults with limited English proficiency,^31^ Indigenous Australian youth^29^) showed a medium to high effect size of mobile app interventions (SMD, 0.63; 95% CI, 0.43 to 0.83) (Table 3). In addition, participants receiving ongoing psychotherapy or psychotropic medications showed significantly smaller effect sizes than those who did not receive any ongoing treatments (psychotherapy: *Q* = 8.60; *P* = .003; medication: *Q* = 11.45; *P* = .001). Furthermore, participants with Western ethnicities showed a significantly smaller effect size than those with non-Western ethnicities (*Q* = 23.69; *P* < .001). In contrast, the continuous variables of participant’s mean age and dropout rate, as well as the trichotomous variables of the mean age in the 20s, 30s, and 40s (*Q* = 5.17; *P* = .08), were not associated with moderating effect sizes (Table 3; eTable 2 in Supplement 1). Additional analysis of participants with formal depression diagnosis vs those with self-reported moderate and severe depressive symptoms also showed no significant group difference (*Q* = 2.11; *P* = .15) (Table 3).

**Table 3.** Subgroup Analyses Based on Population and Study Design Characteristics

Characteristic	Apps, No.	Sample size, intervention/control, No.	Meta-analysis		Heterogeneity			Between-group tests	
			Hedge *g (*95% CI)	*P* value	*Q*	*P* value	*I*^2^, %	*Q*	*P* value
**Population-level characteristics**										
Marginalized group	4	212/202	0.632 (0.433 to 0.831)	<.001	2.35	.50	0.0	NA	NA
Receipt of ongoing psychotherapy									
Yes	5	193/178	0.233 (0.024 to 0.442)	.03	10.16	.04	60.6	8.60	.003
No	11	563/536	0.595 (0.473 to 0.717)	<.001	27.42	.002	63.5		
Received ongoing medication									
Yes	12	406/378	0.333 (0.189 to 0.477)	<.001	33.02	.001	66.7	11.45	.001
No	4	350/336	0.698 (0.543 to 0.852	<.001	1.71	.64	0.0		
Western ethnicity									
Yes	9	276/251	0.155 (−0.020 to 0.330)	.08	18.18	.02	56.0	23.69	<.001
No	7	480/463	0.700 (0.568 to 0.831)	<.001	4.31	.64	0.0		
Mean age, y									
20s	7	504/496	0.508 (0.381 to 0.635)	<.001	27.81	<.001	78.4	5.17	.08
30s	2	61/51	0.818 (0.424 to 1.212	<.001	1.39	.24	28.0		
40s	5	171/147	0.310 (0.084 to 0.536)	.007	5.74	.22	30.3		
Depression diagnosis									
Formal diagnosis	5	366/349	0.424 (0.275 to 0.574)	<.001	22.78	<.001	82.4	2.11	.15
Self-reported symptoms	11	390/365	0.580 (0.432 to 0.729)	<.001	21.29	.02	53.0		
**Study design characteristics**										
Publication date									
≤2019	10	262/233	0.433 (0.250 to 0.615)	<.001	16.33	.06	44.9	0.85	.36
>2019	6	494/481	0.538 (0.409 to 0.667)	<.001	29.01	<.001	82.8		
Use of rewards									
Yes	10	467/420	0.719 (0.582 to 0.857)	<.001	9.70	.38	7.3	34.86	<.001
No	3	182/191	−0.020 (−0.223 to 0.183)	.85	0.24	.89	0.0		
Intervention duration, wk									
<8	6	251/236	0.770 (0.585 to 0.955)	<.001	5.32	.38	6.0	8.15	.004
≥8	9	445/409	0.433 (0.296 to 0.571)	<.001	24.06	.002	66.8		
**Intervention-level characteristics**										
With ongoing professional support									
Yes	9	459/464	0.379 (0.248 to 0.511)	<.001	31.31	<.001	74.4	9.44	.002
No	7	297/250	0.724 (0.548 to 0.900)	<.001	5.44	.49	0.0		
In-app notifications									
Yes	6	337/339	0.445 (0.290 to 0.599)	<.001	20.03	.001	75.0	5.24	.02
No	8	347/303	0.705 (0.544 to 0.865)	<.001	7.58	.37	7.7		
Involved CBT modules									
Yes	12	684/668	0.492 (0.383 to 0.602)	<.001	45.19	<.001	75.7	0.49	.48
No	4	72/46	0.637 (0.248 to 1.025)	.001	0.50	.92	0.0		
Involved BA modules									
Yes	9	459/437	0.463 (0.328 to 0.597)	<.001	26.06	.001	69.3	0.88	.35
No	7	297/277	0.566 (0.397 to 0.736)	<.001	19.24	.004	68.8		
Involved ACT or mindfulness modules									
Yes	4	193/200	0.271 (0.070 to 0.473)	.008	18.73	<.001	84.0	7.00	.008
No	12	563/514	0.590 (0.467 to 0.714)	<.001	20.45	.04	46.2		
Involved mood-tracking modules									
Yes	8	285/256	0.321 (0.148 to 0.494)	<.001	12.62	.08	44.5	6.73	.009
No	8	471/458	0.610 (0.477 to 0.743)	<.001	26.83	<.001	73.9		

Abbreviations: ACT, acceptance and commitment therapy; BA, behavioral activation; CBT, cognitive behavioral therapy.

### Subgroup Analyses of Study Design Characteristics

Regarding the study-level characteristics, the continuous and categorical variables of publication timing did not show a significant moderating association, but subgroup analyses of studies that offered rewards or incentives (*Q* = 34.86; *P* < .001) showed a significantly larger effect size than studies without rewards (Table 3; eTable 2 in Supplement 1). In addition, interventions delivered for less than 8 weeks were associated with a greater effect size (SMD, 0.77; 95% CI, 0.59-0.96) than those delivered for 8 weeks or longer (SMD, 0.43; 95% CI, 0.30-0.57; *Q* = 8.15; *P* = .004), in which 8 weeks were both mean and median values of the duration of the included studies (Table 3).

### Subgroup Analyses of Components of the Intervention Programs

Subgroup analyses of the interventions with vs without professional support (eg, providing feedback, chat messages, phone calls, and supportive coaching) (*Q* = 9.44; *P* = .002) and those with vs without in-app notifications (SMD, 0.45; 95% CI, 0.29-0.60 vs SMD, 0.71; 95% CI, 0.54-0.87; *Q* = 5.24; *P* = .02) showed significant group differences, with the effect sizes favoring apps without professional support and without in-app notifications (Table 3). Regarding therapeutic strategies, there were no significant differences in the effect sizes of interventions with vs without cognitive behavioral therapy (CBT) (*Q* = 0.49; *P* = .48) and behavioral activation (BA) modules (*Q* = 0.88; *P* = .35); however, significant differences were observed when comparing interventions with vs without acceptance and commitment therapy (ACT) or mindfulness (*Q* = 7.00; *P* = .008) and mood tracker (*Q* = 6.73; *P* = .009) modules (Table 3).

## Discussion

To our knowledge, this systematic review and meta-analysis is the first to examine individuals with moderate to severe depression to show the efficacy of mobile app interventions. We found a significant medium effect size, consistent with previous meta-analysis results.^22,23,24^ The significant treatment efficacy of app-based interventions compared with active and inactive controls suggests the potential of mobile app interventions as an alternative to conventional psychotherapy, with further merits in accessibility, financial affordability, and safety from stigma.

### Population Characteristics

First, our study demonstrated the potential efficacy of app interventions for individuals from marginalized groups. Given the heightened stigma faced by these populations and their limited access to mental health services,^48,49^ our findings highlight the importance of further development and refinement of mobile-based, non–face-to-face treatments tailored to socially disadvantaged populations.

Second, we observed significant reductions in depression severity for participants who received ongoing psychological or medication treatments and those who did not. However, the effect sizes were more pronounced in participants not receiving ongoing treatment. A study by Park et al,^21^ consistent with our findings, proposed a ceiling effect as a plausible explanation, indicating that individuals already receiving conventional treatments may have less room for symptom improvement. Nonetheless, it is noteworthy that our results show significant treatment effect sizes evident in both groups, establishing the efficacy of app-based interventions as standalone treatments and adjuncts to conventional therapies, contrary to concerns raised in previous reviews.^23,26^

Third, participants with non-Western ethnicities obtained a significant treatment efficacy associated with app-based interventions, while participants with Western ethnicities did not. This discrepancy could be attributed to the higher mental disorder stigma in non-Western cultures, leading to a greater preference for mHealth interventions,^48,50^ thereby positively contributing to symptom improvement.^51^ Future research should examine the interaction between stigma and treatment outcomes of app-based interventions in both Western and non-Western cultures.

Fourth, the mean age of the study participants was not a significant moderator of the treatment efficacy associated with app-based interventions. However, the studies included in this review involved participants with a mean age ranging from their 20s to 40s, so further studies with adults aged 50 years and older are necessary to generalize our findings to older populations.

Fifth, participants with formal diagnosis vs self-reported depressive symptoms showed no significant group difference in the efficacy of the app interventions. This finding suggests that there is broader applicability of mobile app interventions even for the clinical population.

### Study Design Characteristics

Our analyses showed no significant difference in treatment efficacy between studies published in or before and after 2019. However, an interesting finding emerged that studies that offered monetary rewards yielded a significantly greater effect size for app-based interventions than those without such incentives. Consistent with a previous meta-analysis,^52^ this finding implies that rewards can improve treatment outcomes of and adherence to app-based interventions for depression. Future research should explore ways to incorporate nonmonetary rewards into the app, as this may further optimize the effectiveness of app-based interventions for depression.

In addition, we found that shorter interventions were associated with greater treatment efficacy, although previous meta-analyses have not found significant associations between the length and outcome of interventions.^21,22,53,54^ Other studies have reported that longer interventions were not associated with greater benefits than shorter ones.^55,56^ Therefore, our findings recommend developing app-based programs that are shorter than 8 weeks to optimize the effectiveness of depression treatment and to reduce the financial and psychological burdens on the participants.

### Components of the Intervention Programs

First, our study supported the findings of the systematic review of Firth et al,^22^ demonstrating significant treatment effect sizes of app-based programs both with and without professional support, with the effect size favoring studies without professional support. This finding highlights the potential for app-based interventions to produce positive outcomes for individuals struggling with moderate and severe depression, even without professional support. Some studies contradicted our results,^20,27^ which may have been due to our study limiting the depression severity to moderate and severe.

Second, in contrast to previous reviews^20,27^ and counterintuitively, mHealth apps with in-app reminders had a smaller effect size than those without. However, it is worth noting that researchers using apps without in-app reminders provided weekly notifications through text messages or email reports. Therefore, our results highlight the importance of avoiding sole reliance on in-app notifications when prescribing mHealth apps in a clinical setting. Instead, in-person reminders may be more effective in motivating patients to continue using the evidence-based mHealth apps.

Third, there was no significant difference in the efficacy between apps with and without CBT or BA modules. However, apps with ACT or mindfulness or mood-tracking modules had significantly smaller effect sizes than those without. A previous RCT showed that a BA-based smartphone app had greater efficacy for individuals with severe depression, whereas a mindfulness-based app was more beneficial for those with mild depression.^57^ Based on our findings, we propose CBT- and BA-based apps as the foremost recommended interventions for effectively addressing moderate to severe depression. Nevertheless, comprehensive research is necessary to discern the optimal therapeutic approaches for varying levels of depression severity, thus enhancing the treatment efficacy by tailoring app-based interventions accordingly.

### Limitations

This study has several limitations. First, while the sample consisted of individuals with moderate to severe depressive symptoms or a depression diagnosis, it is possible that some participants were diagnosed with mild levels of depressive symptoms. However, the proportion of participants with only mild depressive symptoms is likely to be small, given that those diagnosed with depressive disorders had moderate and severe baseline depression scores,^41,43,45^ with the exception of 1 study.^33^ Future research should include participants with mild depression and explore the association between symptom severity and possible moderators of treatment efficacy. Second, despite restrictive eligibility criteria, significant heterogeneity was observed among the included studies. However, the heterogeneity was substantially reduced when the studies were stratified based on the types of control groups and the use of rewards, implying that the variations in study design were the primary sources of heterogeneity. Third, the literature search was limited to English-language publications, which might have limited a more comprehensive review. Fourth, a relatively small number of studies were included in this analysis (13 studies), although this was an inevitable consequence of limiting the study population to individuals with moderate and severe depression. Fifth, using univariate analysis and summary data (eg, the mean age) for the subgroup analyses might have prevented a detailed understanding of the moderating associations of participant characteristics. Future research should conduct multivariate analyses across more studies and use individual participant data to support the current findings.

## Conclusions

In this systematic review and meta-analysis of the efficacy associated with app-based interventions for moderate to severe depression, a significant reduction was found in depression severity associated with use of app interventions. In addition, our study underscores the importance of proactively considering population and study design characteristics and app-based program components to further improve the effectiveness of mHealth apps. In particular, the user’s cultural background, incorporation of in-app incentives and in-person reminders, duration of app use, and CBT- and BA-based modules should be considered for more effective design and application of mobile app interventions for individuals with moderate to severe depression. These findings are expected to provide developers and researchers in the rapidly evolving field of mHealth with practical insights into the development, prescription, and implementation of app-based depression interventions.

## Notes

Supplement 1. **eTable 1.** Egger Regression Test for the Assessment of Publication Bias **eTable 2.** Results of Exploratory Post Hoc Meta-Regression Analyses **eMethods 1.** Keywords Used in Search of Studies **eMethods 2.** Inclusion and Exclusion Criteria **eFigure 1.** Traffic Light Plot for Risk of Bias of Each Included Study **eFigure 2.** The Overall Risk of Bias Summary of the Included Studies **eFigure 3.** Sensitivity Analysis by the Leave-One-Out Method **eFigure 4.** Funnel Plot Assessing Publication Bias for the Efficacy of Mobile App Intervention for Depression **eFigure 5.** Duval and Tweedie Trim-and-Fill Funnel Plot

Supplement 2.
Data Sharing Statement

## References

[1] World Health Organization. Depressive disorder (depression): fact sheets. Accessed June 13, 2023. https://www.who.int/news-room/fact-sheets/detail/depression

[2] American Psychiatric Association. Diagnostic and Statistical Manual of Mental Disorders. 5th ed. American Psychiatric Association; 2013.

[3] Tolentino JC, Schmidt SL. *DSM-5* criteria and depression severity: implications for clinical practice. Front Psychiatry. 2018;9:450. doi:10.3389/fpsyt.2018.00450 30333763 PMC6176119

[4] Hawton K, Casañas I Comabella C, Haw C, Saunders K. Risk factors for suicide in individuals with depression: a systematic review. J Affect Disord. 2013;147(1-3):17-28. doi:10.1016/j.jad.2013.01.004 23411024

[5] Cuijpers P, Noma H, Karyotaki E, Cipriani A, Furukawa TA. Effectiveness and acceptability of cognitive behavior therapy delivery formats in adults with depression: a network meta-analysis. JAMA Psychiatry. 2019;76(7):700-707. doi:10.1001/jamapsychiatry.2019.0268 30994877 PMC6583673

[6] Zhang L, Liu X, Tong F, . Cognitive behavioral therapy for anxiety and depression in cancer survivors: a meta-analysis. Sci Rep. 2022;12(1):21466. doi:10.1038/s41598-022-25068-7 36509786 PMC9744858

[7] Strauss C, Bibby-Jones A-M, Jones F, . Clinical effectiveness and cost-effectiveness of supported mindfulness-based cognitive therapy self-help compared with supported cognitive behavioral therapy self-help for adults experiencing depression: the Low-Intensity Guided Help Through Mindfulness (LIGHTMind) randomized clinical trial. JAMA Psychiatry. 2023;80(5):415-424. doi:10.1001/jamapsychiatry.2023.0222 36947058 PMC10034662

[8] Oberoi S, Yang J, Woodgate RL, . Association of mindfulness-based interventions with anxiety severity in adults with cancer: a systematic review and meta-analysis. JAMA Netw Open. 2020;3(8):e2012598. doi:10.1001/jamanetworkopen.2020.12598 32766801 PMC7414391

[9] Wright JH, Owen J, Eells TD, . Effect of computer-assisted cognitive behavior therapy vs usual care on depression among adults in primary care: a randomized clinical trial. JAMA Netw Open. 2022;5(2):e2146716. doi:10.1001/jamanetworkopen.2021.46716 35142833 PMC8832170

[10] Andrade LH, Alonso J, Mneimneh Z, . Barriers to mental health treatment: results from the WHO World Mental Health surveys. Psychol Med. 2014;44(6):1303-1317. doi:10.1017/S0033291713001943 23931656 PMC4100460

[11] Mojtabai R, Olfson M, Sampson NA, . Barriers to mental health treatment: results from the National Comorbidity Survey Replication. Psychol Med. 2011;41(8):1751-1761. doi:10.1017/S0033291710002291 21134315 PMC3128692

[12] Evans-Lacko S, Aguilar-Gaxiola S, Al-Hamzawi A, . Socio-economic variations in the mental health treatment gap for people with anxiety, mood, and substance use disorders: results from the WHO World Mental Health (WMH) surveys. Psychol Med. 2018;48(9):1560-1571. doi:10.1017/S0033291717003336 29173244 PMC6878971

[13] Wasil AR, Gillespie S, Schell T, Lorenzo-Luaces L, DeRubeis RJ. Estimating the real-world usage of mobile apps for mental health: development and application of two novel metrics. World Psychiatry. 2021;20(1):137-138. doi:10.1002/wps.20827 33432761 PMC7801857

[14] Biagianti B, Hidalgo-Mazzei D, Meyer N. Developing digital interventions for people living with serious mental illness: perspectives from three mHealth studies. Evid Based Ment Health. 2017;20(4):98-101. doi:10.1136/eb-2017-102765 29025862 PMC5750413

[15] Sun S, Lin D, Goldberg S, . A mindfulness-based mobile health (mHealth) intervention among psychologically distressed university students in quarantine during the COVID-19 pandemic: a randomized controlled trial. J Couns Psychol. 2022;69(2):157-171. doi:10.1037/cou0000568 34264696 PMC8760365

[16] Rowland SP, Fitzgerald JE, Holme T, Powell J, McGregor A. What is the clinical value of mHealth for patients? NPJ Digit Med. 2020;3(1):4. doi:10.1038/s41746-019-0206-x 31970289 PMC6957674

[17] Ardi Z, Sukmawati I, Ifdil I, . Exploring the acceptability of internet-based mental health mobile app services using network psychometrics analysis. J Phys Conf Ser. 2018;1114:012106. doi:10.1088/1742-6596/1114/1/012106

[18] Chen PV, Helm A, Caloudas SG, . Evidence of phone vs video-conferencing for mental health treatments: a review of the literature. Curr Psychiatry Rep. 2022;24(10):529-539. doi:10.1007/s11920-022-01359-8 36053400 PMC9437398

[19] Wu A, Scult MA, Barnes ED, Betancourt JA, Falk A, Gunning FM. Smartphone apps for depression and anxiety: a systematic review and meta-analysis of techniques to increase engagement. NPJ Digit Med. 2021;4(1):20. doi:10.1038/s41746-021-00386-8 33574573 PMC7878769

[20] Linardon J, Cuijpers P, Carlbring P, Messer M, Fuller-Tyszkiewicz M. The efficacy of app-supported smartphone interventions for mental health problems: a meta-analysis of randomized controlled trials. World Psychiatry. 2019;18(3):325-336. doi:10.1002/wps.20673 31496095 PMC6732686

[21] Park C, Zhu J, Ho Chun Man R, . Smartphone applications for the treatment of depressive symptoms: a meta-analysis and qualitative review. Ann Clin Psychiatry. 2020;32(1):48-68. 31675391

[22] Firth J, Torous J, Nicholas J, . The efficacy of smartphone-based mental health interventions for depressive symptoms: a meta-analysis of randomized controlled trials. World Psychiatry. 2017;16(3):287-298. doi:10.1002/wps.20472 28941113 PMC5608852

[23] Weisel KK, Fuhrmann LM, Berking M, Baumeister H, Cuijpers P, Ebert DD. Standalone smartphone apps for mental health-a systematic review and meta-analysis. NPJ Digit Med. 2019;2(1):118. doi:10.1038/s41746-019-0188-8 31815193 PMC6889400

[24] Serrano-Ripoll MJ, Zamanillo-Campos R, Fiol-DeRoque MA, Castro A, Ricci-Cabello I. Impact of smartphone app-based psychological interventions for reducing depressive symptoms in people with depression: systematic literature review and meta-analysis of randomized controlled trials. JMIR Mhealth Uhealth. 2022;10(1):e29621. doi:10.2196/29621 35084346 PMC8832272

[25] Josephine K, Josefine L, Philipp D, David E, Harald B. Internet- and mobile-based depression interventions for people with diagnosed depression: a systematic review and meta-analysis. J Affect Disord. 2017;223:28-40. doi:10.1016/j.jad.2017.07.021 28715726

[26] Lu S-C, Xu M, Wang M, . Effectiveness and minimum effective dose of app-based mobile health interventions for anxiety and depression symptom reduction: systematic review and meta-analysis. JMIR Ment Health. 2022;9(9):e39454. doi:10.2196/39454 36069841 PMC9494214

[27] Kim SK, Lee M, Jeong H, Jang YM. Effectiveness of mobile applications for patients with severe mental illness: a meta-analysis of randomized controlled trials. Jpn J Nurs Sci. 2022;19(3):e12476. doi:10.1111/jjns.12476 35174976

[28] Page MJ, McKenzie JE, Bossuyt PM, . The PRISMA 2020 statement: an updated guideline for reporting systematic reviews. Int J Surg. 2021;88:105906. doi:10.1016/j.ijsu.2021.105906 33789826

[29] Tighe J, Shand F, Ridani R, Mackinnon A, De La Mata N, Christensen H. Ibobbly mobile health intervention for suicide prevention in Australian Indigenous youth: a pilot randomised controlled trial. BMJ Open. 2017;7(1):e013518. doi:10.1136/bmjopen-2016-013518 28132007 PMC5278278

[30] Schardt C, Adams MB, Owens T, Keitz S, Fontelo P. Utilization of the PICO framework to improve searching PubMed for clinical questions. BMC Med Inform Decis Mak. Published online June 15, 2007. doi:10.1186/1472-6947-7-16 17573961 PMC1904193

[31] Dahne J, Collado A, Lejuez CW, . Pilot randomized controlled trial of a Spanish-language behavioral activation mobile app (¡Aptívate!) for the treatment of depressive symptoms among United States Latinx adults with limited English proficiency. J Affect Disord. 2019;250:210-217. doi:10.1016/j.jad.2019.03.009 30870770 PMC6461510

[32] Dahne J, Lejuez CW, Diaz VA, . Pilot randomized trial of a self-help behavioral activation mobile app for utilization in primary care. Behav Ther. 2019;50(4):817-827. doi:10.1016/j.beth.2018.12.003 31208690 PMC6582985

[33] Tønning ML, Faurholt-Jepsen M, Frost M, . The effect of smartphone-based monitoring and treatment on the rate and duration of psychiatric readmission in patients with unipolar depressive disorder: the RADMIS randomized controlled trial. J Affect Disord. 2021;282:354-363. doi:10.1016/j.jad.2020.12.141 33421863

[34] Sterne JAC, Savović J, Page MJ, . ROB 2: a revised tool for assessing risk of bias in randomised trials. BMJ. 2019;366:l4898. doi:10.1136/bmj.l4898 31462531

[35] Sterne JA, Egger M. Regression methods to detect publication and other bias in meta-analysis. In: Rothstein HR, Sutton AJ, Borenstein M, eds. Publication Bias in Meta-Analysis: Prevention, Assessment and Adjustments. Wiley; 2005:99-110. doi:10.1002/0470870168.ch6

[36] Duval S, Tweedie R. Trim and fill: a simple funnel-plot-based method of testing and adjusting for publication bias in meta-analysis. Biometrics. 2000;56(2):455-463. doi:10.1111/j.0006-341X.2000.00455.x 10877304

[37] Higgins JP, Thompson SG, Deeks JJ, Altman DG. Measuring inconsistency in meta-analyses. BMJ. 2003;327(7414):557-560. doi:10.1136/bmj.327.7414.557 12958120 PMC192859

[38] Stiles-Shields C, Montague E, Kwasny MJ, Mohr DC. Behavioral and cognitive intervention strategies delivered via coached apps for depression: pilot trial. Psychol Serv. 2019;16(2):233-238. doi:10.1037/ser0000261 30407055 PMC6499699

[39] Chan CS, Wong CY, Branda Y, . Treating depression with a smartphone-delivered self-help cognitive behavioral therapy for insomnia: a parallel-group randomized controlled trial. Psychol Med. 2023;53(5):1799-1813. doi:10.1017/S0033291721003421 37310329

[40] Guo Y, Hong YA, Cai W, . Effect of a WeChat-based intervention (Run4Love) on depressive symptoms among people living with HIV in China: a randomized controlled trial. J Med Internet Res. 2020;22(2):e16715. doi:10.2196/16715 32044751 PMC7058168

[41] Hur J-W, Kim B, Park D, Choi S-W. A scenario-based cognitive behavioral therapy mobile app to reduce dysfunctional beliefs in individuals with depression: a randomized controlled trial. Telemed J E Health. 2018;24(9):710-716. doi:10.1089/tmj.2017.0214 29323626

[42] Kageyama K, Kato Y, Mesaki T, . Effects of video viewing smartphone application intervention involving positive word stimulation in people with subthreshold depression: a pilot randomized controlled trial. J Affect Disord. 2021;282:74-81. doi:10.1016/j.jad.2020.12.104 33401126

[43] Mantani A, Kato T, Furukawa TA, . Smartphone cognitive behavioral therapy as an adjunct to pharmacotherapy for refractory depression: randomized controlled trial. J Med Internet Res. 2017;19(11):e373. doi:10.2196/jmir.8602 29101095 PMC5695656

[44] O’Toole MS, Arendt MB, Pedersen CM. Testing an app-assisted treatment for suicide prevention in a randomized controlled trial: effects on suicide risk and depression. Behav Ther. 2019;50(2):421-429. doi:10.1016/j.beth.2018.07.007 30824256

[45] Raevuori A, Vahlberg T, Korhonen T, Hilgert O, Aittakumpu-Hyden R, Forman-Hoffman V. A therapist-guided smartphone app for major depression in young adults: a randomized clinical trial. J Affect Disord. 2021;286:228-238. doi:10.1016/j.jad.2021.02.007 33743385

[46] Wong VWH, Ho FYY, Shi NK, . Smartphone-delivered multicomponent lifestyle medicine intervention for depressive symptoms: a randomized controlled trial. J Consult Clin Psychol. 2021;89(12):970-984. doi:10.1037/ccp0000695 35025538

[47] Bech P, Timmerby N, Martiny K, Lunde M, Soendergaard S. Psychometric evaluation of the Major Depression Inventory (MDI) as depression severity scale using the LEAD (Longitudinal Expert Assessment of All Data) as index of validity. BMC Psychiatry. 2015;15(1):190. doi:10.1186/s12888-015-0529-3 26242577 PMC4526416

[48] Wong EC, Collins RL, Cerully J, Seelam R, Roth B. Racial and ethnic differences in mental illness stigma and discrimination among Californians experiencing mental health challenges. Rand Health Q. 2017;6(2):6. 28845344 10.7249/rhq-06-02-06 PMC5568160

[49] Eylem O, de Wit L, van Straten A, . Stigma for common mental disorders in racial minorities and majorities a systematic review and meta-analysis. BMC Public Health. 2020;20(1):879. doi:10.1186/s12889-020-08964-3 32513215 PMC7278062

[50] Krendl AC, Pescosolido BA. Countries and cultural differences in the stigma of mental illness: the East–West divide. J Cross Cult Psychol. 2020;51(2):149-167. doi:10.1177/0022022119901297

[51] Swift JK, Callahan JL. The impact of client treatment preferences on outcome: a meta-analysis. J Clin Psychol. 2009;65(4):368-381. doi:10.1002/jclp.20553 19226606

[52] Khazanov GK, Morris PE, Beed A, . Do financial incentives increase mental health treatment engagement: a meta-analysis. J Consult Clin Psychol. 2022;90(6):528-544. doi:10.1037/ccp0000737 35771513 PMC10603786

[53] Liu JJ, Ein N, Forchuk C, . A meta-analysis of internet-based cognitive behavioral therapy for military and veteran populations. BMC Psychiatry. 2023;23(1):223. doi:10.1186/s12888-023-04668-1 37013501 PMC10068715

[54] Heber E, Ebert DD, Lehr D, . The benefit of web- and computer-based interventions for stress: a systematic review and meta-analysis. J Med Internet Res. 2017;19(2):e32. doi:10.2196/jmir.5774 28213341 PMC5336602

[55] Juul S, Jakobsen JC, Jørgensen CK, Poulsen S, Sørensen P, Simonsen S. The difference between shorter- versus longer-term psychotherapy for adult mental health disorders: a systematic review with meta-analysis. BMC Psychiatry. 2023;23(1):438. doi:10.1186/s12888-023-04895-6 37328755 PMC10273498

[56] Lorentzen S, Ruud T, Fjeldstad A, Høglend P. Comparison of short- and long-term dynamic group psychotherapy: randomised clinical trial. Br J Psychiatry. 2013;203(3):280-287. doi:10.1192/bjp.bp.112.113688 24029539

[57] Ly KH, Trüschel A, Jarl L, . Behavioural activation versus mindfulness-based guided self-help treatment administered through a smartphone application: a randomised controlled trial. BMJ Open. 2014;4(1):e003440. doi:10.1136/bmjopen-2013-003440 24413342 PMC3902198

