# Supplementary material for: App-Based Interventions for Moderate to Severe Depression: A Systematic Review and Meta-Analysis
Source: JAMA Netw Open. 2023 Nov 20;6(11):e2344120. doi: 10.1001/jamanetworkopen.2023.44120 (PMC10660171; doi:10.1001/jamanetworkopen.2023.44120)
Supplement: Supplement 1. — eTable 1. Egger Regression Test for the Assessment of Publication Bias eTable 2. Results of Exploratory Post Hoc Meta-Regression Analyses eMethods 1. Keywords Used in Search of Studies eMethods 2. Inclusion and Exclusion Criteria eFigure 1. Traffic Light Plot for Risk of Bias of Each Included Study eFigure 2. The Overall Risk of Bias Summary of the Included Studies eFigure 3. Sensitivity Analysis by the Leave-One-Out Method eFigure 4. Funnel Plot Assessing Publication Bias for the Efficacy of Mobile App Intervention for Depression eFigure 5. Duval and Tweedie Trim-and-Fill Funnel Plot [file jamanetwopen-e2344120-s001.pdf]

Supplemental Online Content

Bae H, Shin H, Ji H, Kwon JS, Kim H, Hur J. App-based interventions for moderate to severe depression: a systematic review and meta-analysis. *JAMA Netw Open*. 2023;6(11):e2344120. doi:10.1001/jamanetworkopen.2023.44120

eTable 1. Egger Regression Test for the Assessment of Publication Bias
eTable 2. Results of Exploratory Post Hoc Meta-Regression Analyses
eMethods 1. Keywords Used in Search of Studies
eMethods 2. Inclusion and Exclusion Criteria
eFigure 1. Traffic Light Plot for Risk of Bias of Each Included Study
eFigure 2. The Overall Risk of Bias Summary of the Included Studies
eFigure 3. Sensitivity Analysis by the Leave-One-Out Method
eFigure 4. Funnel Plot Assessing Publication Bias for the Efficacy of Mobile App Intervention for Depression
eFigure 5. Duval and Tweedie Trim-and-Fill Funnel Plot

This supplemental material has been provided by the authors to give readers additional information about their work.

eTable 1. Egger Regression Test for the Assessment of Publication Bias

SMD	Coefficient	*SE*	*t*	*P*	95% CI	
Slope	0.469	0.216	2.17	.05	0.006	0.933
Bias	0.175	1.005	0.17	.87	−1.981	2.330

© 2023 Bae H et al. *JAMA Network Open.*

eTable 2. Results of Exploratory Post Hoc Meta-Regression Analyses

Continuous variables	Number of apps	Sample size (intervention /control)	*β*	95% CI		*SE*	*P*
Mean age	14	736/674	−0.001	−0.032	0.030	0.014	.96
Publication year	16	756/684	−0.032	−0.198	0.134	0.077	.69
Dropout rate	16	756/684	−0.009	−0.021	0.003	0.006	.14

© 2023 Bae H et al. *JAMA Network Open.*

eMethods 1. Keywords Used in Search of Studies

→ For PubMed:

('depression'[Title/Abstract] OR 'depressive symptoms'[Title/Abstract] OR 'depressive disorder'[Title/Abstract]) AND ('mobile application'[Title/Abstract] OR 'smartphone application'[Title/Abstract] OR mobile[Title/Abstract] OR smartphone[Title/Abstract] OR iphone[Title/Abstract] OR android[Title/Abstract] OR app[Title/Abstract]) AND ('randomized controlled trial'[Title/Abstract] OR randomized[Title/Abstract] OR randomised[Title/Abstract])

→ For Embase:

('depression':ab,ti OR 'depressive symptoms':ab,ti OR 'depressive disorder':ab,ti) AND ('mobile application':ti,ab OR 'smartphone application':ti,ab OR mobile:ti,ab OR smartphone:ti,ab OR iphone:ti,ab OR android:ti,ab OR app:ti,ab) AND ('randomized controlled trial':ti,ab OR randomized:ti,ab OR randomised:ti,ab)

→ For PsycINFO:

(TI depression OR AB depression OR TI "depressive symptoms" OR AB "depressive symptoms" OR TI "depressive disorder" OR AB "depressive disorder") AND (TI "mobile applications" OR AB "mobile applications" OR TI "smartphone applications" OR AB "smartphone applications" OR TI "mobile app" OR AB "mobile app" OR TI "smartphone app" OR AB "smartphone app" OR TI mobile OR AB mobile OR TI smartphone OR AB smartphone OR TI iPhone OR AB iPhone OR TI Android OR AB Android OR TI app OR AB app) AND (TI "randomized controlled trial" OR AB "randomized controlled trial" OR TI randomized OR AB randomized OR TI randomised or AB randomised)

© 2023 Bae H et al. *JAMA Network Open.*

eMethods 2. Inclusion and Exclusion Criteria

Inclusion criteria

Participants: Adults aged 18 years or older with the symptoms of unipolar depression, determined by formal diagnosis or achieving a minimum cut-off score for moderate severity on a validated depression rating scale. There were no restrictions on sex, ethnicity, and nationality.

Intervention: Any therapeutic interventions aiming at ameliorating mental health and well-being delivered via mobile applications on smartphones and/or tablets. No restrictions were placed on the therapeutic orientation employed by the intervention, such as cognitive behavioral therapy (CBT), behavioral activation (BA), positive psychology, or any combination thereof.

Comparator: Control groups could be either inactive (e.g., waitlist control) or active (e.g., treatment-as-usual, attentional control).

Outcomes: Changes in depressive symptoms as measured by the standardized patient- or clinician-rated depression-assessing instruments.

Study Design: Only randomized clinical trials (RCTs) published in English were included.

Exclusion criteria

Exclusion criteria were as follows: (1) were non-randomized studies, dissertations, conference articles, or grey literature, (2) did not have relevant populations (i.e., participants under 18 years of age, with mild or no depressive symptoms, or with bipolar depression and/or postpartum depression[2,3]), (3) did not use mobile application interventions (i.e., web-based eHealth, text-messages or e-mail intervention, or VR exposure therapy), (4) did not report the outcomes of interest (i.e., depressive symptoms), (5) did not report original data (i.e., reviews, meta-analyses, secondary sources, or study protocols), (6) were duplicate articles, and (7) were not published in English.

© 2023 Bae H et al. *JAMA Network Open.*

eFigure 1. Traffic Light Plot for Risk of Bias of Each Included Study[4]

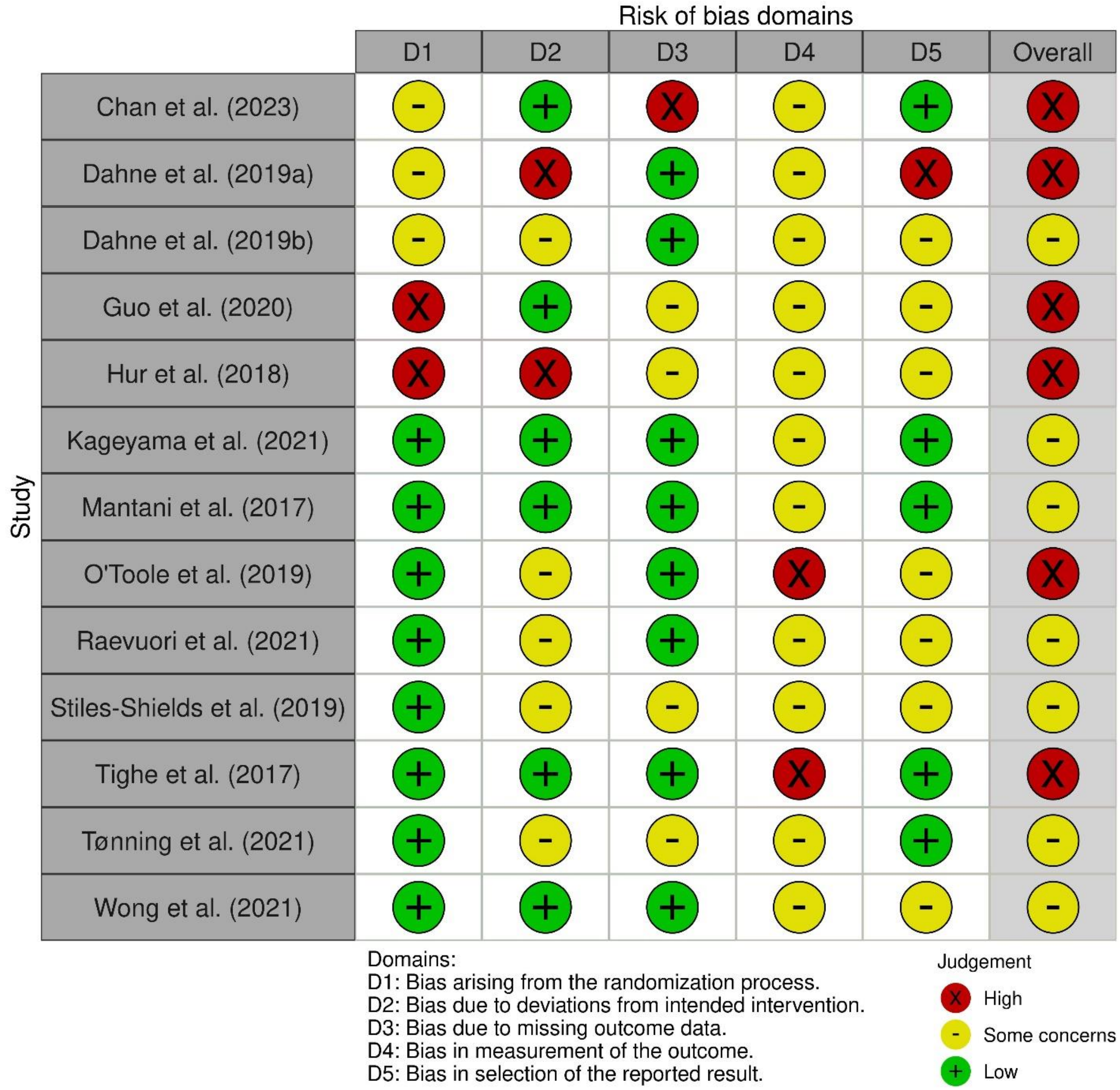

© 2023 Bae H et al. *JAMA Network Open.*

eFigure 2. The Overall Risk of Bias Summary of the Included Studies[4]

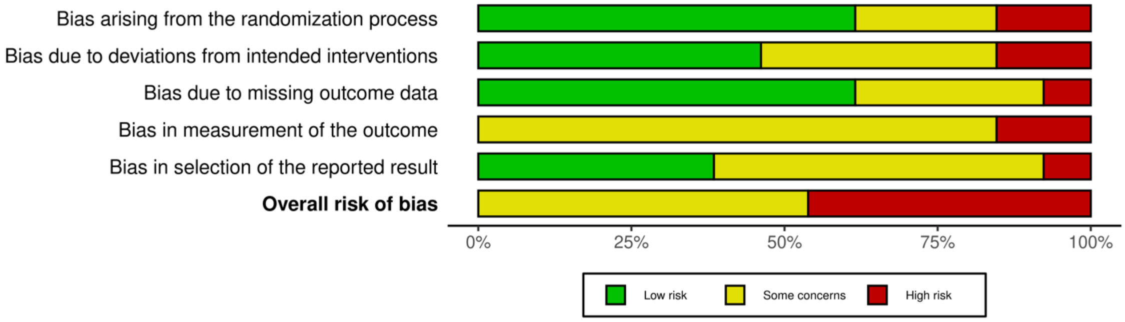

© 2023 Bae H et al. *JAMA Network Open.*

eFigure 3. Sensitivity Analysis by the Leave-One-Out Method

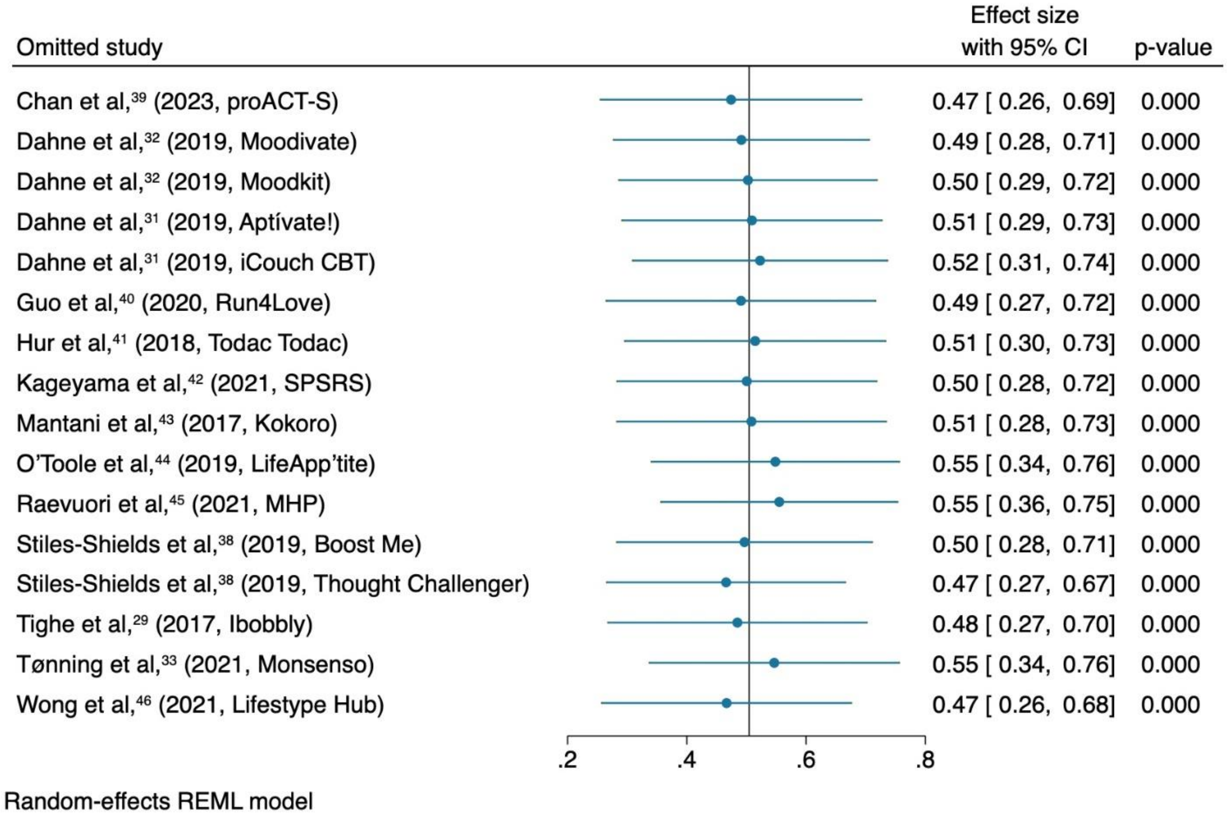

© 2023 Bae H et al. *JAMA Network Open.*

eFigure 4. Funnel Plot Assessing Publication Bias for the Efficacy of Mobile App Intervention for Depression

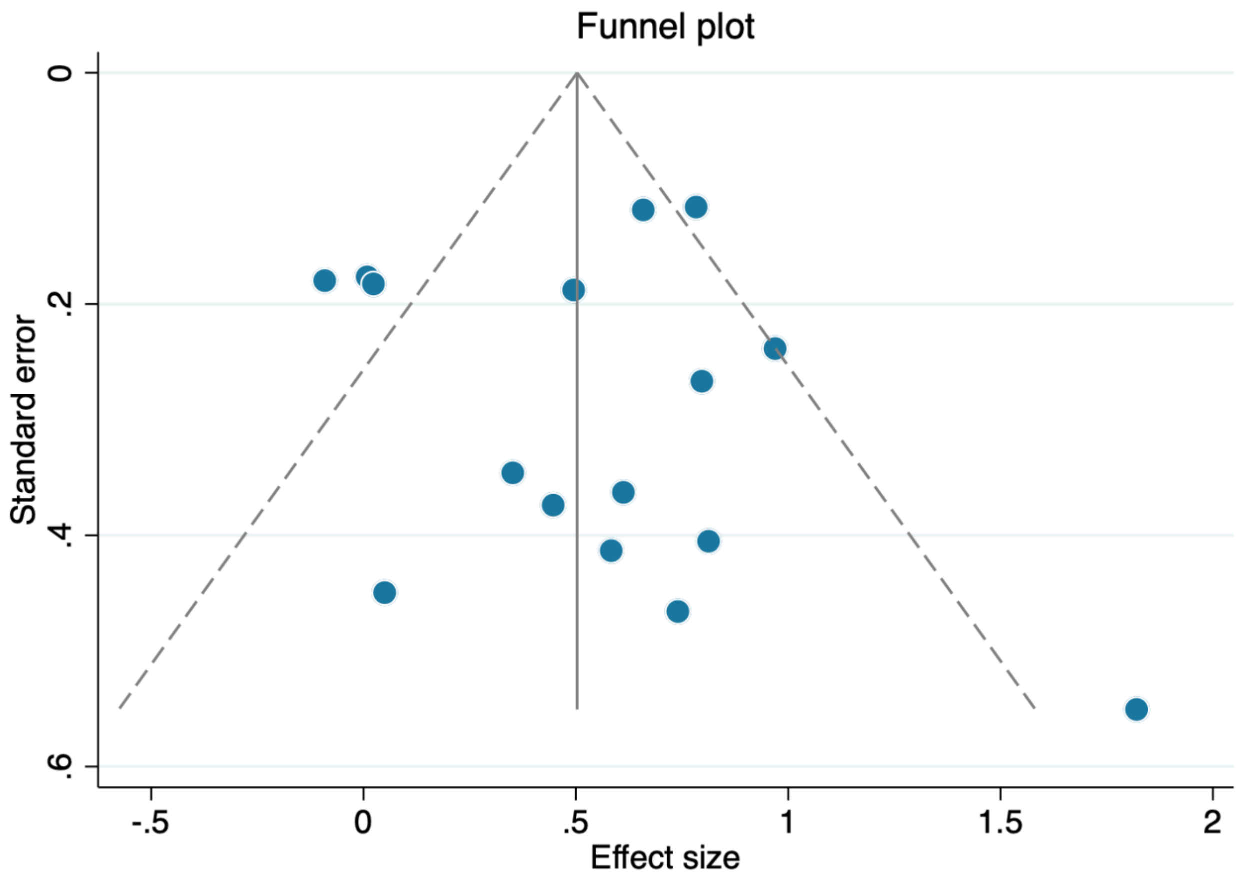

© 2023 Bae H et al. *JAMA Network Open.*

eFigure 5. Duval and Tweedie Trim-and-Fill Funnel Plot

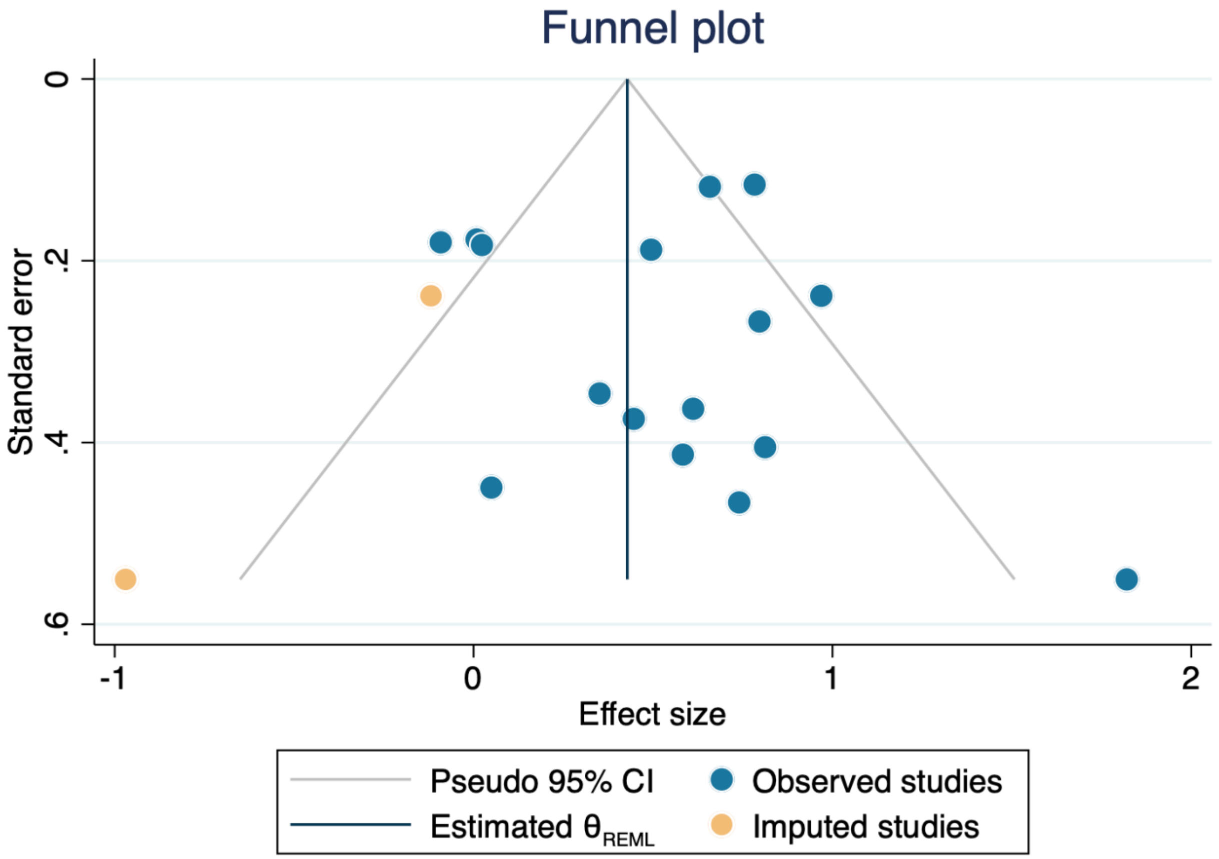

© 2023 Bae H et al. *JAMA Network Open.*

References

1. Page MJ, McKenzie JE, Bossuyt PM, et al. The PRISMA 2020 statement: an updated guideline for reporting systematic reviews. *Int J Surg*. 2021;88:105906. doi:10.1016/j.ijsu.2021.105906
2. Sharma V, Burt VK, Ritchie HL. Bipolar II postpartum depression: detection, diagnosis, and treatment. *Am J Psychiatry*. 2009;166(11):1217-1221. doi:10.1176/appi.ajp.2009.08121902
3. Sharma V, Al-Farayedhi M, Doobay M, Baczynski C. Should all women with postpartum depression be screened for bipolar disorder? *Med Hypotheses*. 2018;118:26-28. doi:10.1016/j.mehy.2018.06.016
4. McGuinness LA, Higgins JPT. Risk-of-bias VISualization (robvis): An R package and Shiny web app for visualizing risk-of-bias assessments. *Res Synth Methods*. 2020/04/26 2020;n/a(n/a)doi:10.1002/jrsm.1411

© 2023 Bae H et al. *JAMA Network Open.*
