# Supplementary material for: App-Based Interventions for Moderate to Severe Depression: A Systematic Review and Meta-Analysis
Source: JAMA Netw Open. 2023 Nov 20;6(11):e2344120. doi: 10.1001/jamanetworkopen.2023.44120 (PMC10660171; doi:10.1001/jamanetworkopen.2023.44120)
Supplement: Supplement 2. — Data Sharing Statement [file jamanetwopen-e2344120-s002.pdf]

Data Sharing Statement

Bae. App-Based Interventions for Moderate to Severe Depression. *JAMA Netw Open*. Published November 20, 2023. doi:10.1001/jamanetworkopen.2023.44120

Data
Data available: No

Additional Information
Explanation for why data not available: This manuscript does not include original data. Data are extracted from literature and are publicly available.
